# Pharmacology of estrogen-related receptors (ERRs) agonists

**DOI:** 10.1039/d6cb00077k

**Published:** 2026-05-29

**Authors:** Mohamed O. Radwan, Soumitra Guin, Thomas P. Burris

**Affiliations:** a Department of Cellular and Systems Pharmacology, University of Florida College of Pharmacy Gainesville Florida 32610 USA burris.thomas@ufl.edu; b University of Florida Genetics Institute Gainesville Florida 32610 USA; c Department of Medicinal Chemistry, University of Florida College of Pharmacy Gainesville Florida 32610 USA

## Abstract

The estrogen-related receptors (ERRs) are a subfamily of orphan nuclear receptors that share high structural homology with classical estrogen receptors but do not bind endogenous estrogens. The three isoforms – ERRα, ERRβ, and ERRγ – exhibit constitutive transcriptional activity and regulate genes involved in mitochondrial biogenesis, oxidative metabolism, and energy homeostasis. ERRα and ERRγ are highly expressed in metabolically active tissues, including the heart, skeletal muscle, and liver, where they play central roles in metabolic adaptation and muscle function. Pharmacological activation of ERRα and ERRγ has therefore emerged as a promising strategy for the treatment of metabolic and muscle-related disorders as well as conditions where mitochondrial function decreases such as aging. This review focuses on the pharmacology of ERRs, their agonists, and the medicinal chemistry strategies underlying their development. Agonists are classified according to chemotype, and the structure–activity relationships (SAR) of each scaffold are analyzed, highlighting key pharmacological strengths and limitations. The chemotypes discussed include acyl hydrazones, 2,5-disubstituted thiophenes, pyrido[1,2-α]pyrimidin-4-ones, 1,2,3-triazoles, bisphenols, and amide-based agonists. Collectively, this work provides a comprehensive framework to guide the rational design of next generation ERRs agonists with improved pharmacological and translational potential.

## Abstract 2

Recent advances in the development of synthetic agonists for the orphan receptors, estrogen-related receptors (ERRs), has revealed their exercise mimetic activity and beneficial effects in skeletal muscle, heart, and brain.

## Graphical abstract

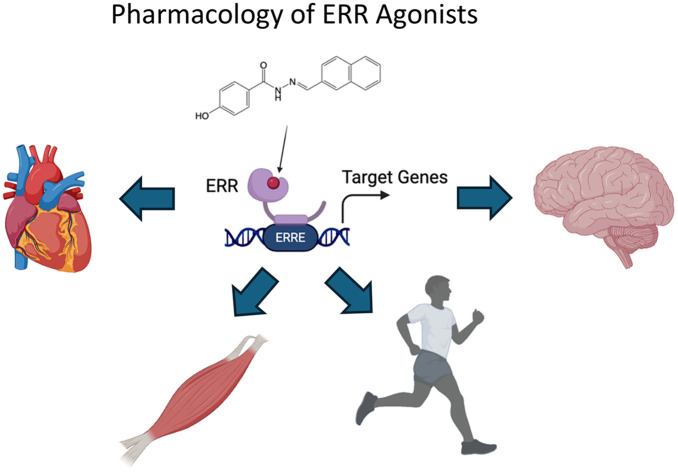

## Introduction

The nuclear receptor (NR) superfamily constitutes 48 ligand-regulated transcription factors in the human genome.^1,2^ NR ligands modulate protein–protein interactions (PPIs) with coregulator proteins (coactivators or corepressors), orchestrating diverse physiological functions, including growth, metabolism, inflammation, reproduction, circadian rhythm, and immunity.^3–5^ The majority of NRs are confirmed to be druggable and valid targets for the treatment of a multitude of non-communicable diseases.^6^

**Fig. 1:**
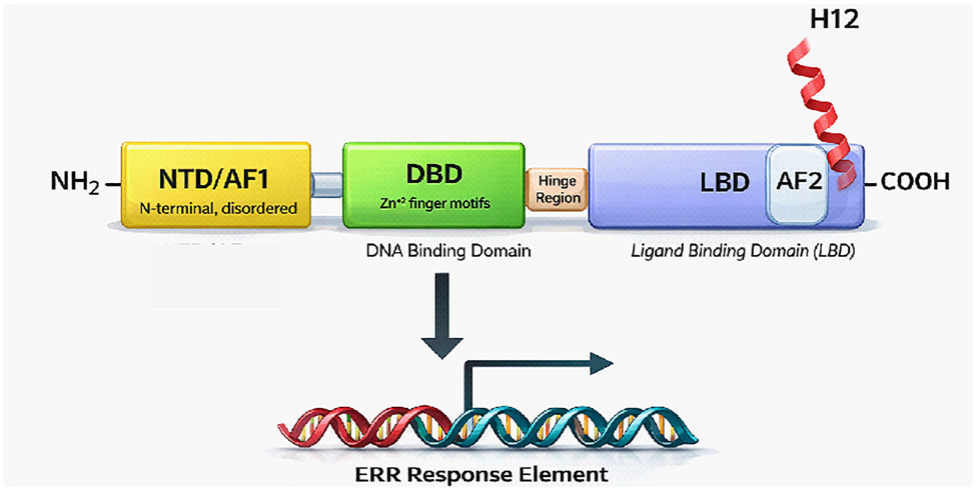
Schematic illustration of the general structure of ERRs.

NRs share considerable homology and conserved structures of four key domains. Additionally, the regions of the receptor are also divided into 5 or 6 distinct regions: A, B, C, D, E, and F.^7,8^ The N-terminal domain, located in the A/B region, possesses a ligand-independent activation function (AF1) and is followed by a central, highly conserved DNA-binding domain (DBD; region C), which enables sequence-specific recognition of DNA in gene promoters (NR response element). This is linked to a less conserved and more flexible D region of the hinge domain that connects the DBD to the E region, which comprises the ligand-binding domain (LBD) and can accommodate hydrophobic small molecules to regulate interactions with cofactors involved in transcriptional regulation. The ligand-dependent activation function (AF-2) resides in the LBD and is closely associated with Helix 12 (H12) at the carboxy-terminus of region E, which undergoes a ligand-dependent conformation change that drives alterations in interactions with transcriptional coactivators and/or corepressors, leading to modulation of target gene expression (Fig. 1).^5,9–11^ Only a limited number of NRs have the F domain, while most NRs have carboxy-terminal regions that end in E. As shown below, H12, the part of LBD that helps form the AF-2 surface, is dynamic and plays different roles depending on the class of ligand bound to the LBD (agonists or inverse agonists).

### Estrogen-related receptors (ERRs)

The estrogen-related receptors (ERRα, ERRβ, and ERRγ, or NR3B1, NR3B2, and NR3B3, respectively) constitute a subfamily of orphan receptors, highly related to NRs in the steroid receptor NR3 subfamily.^9,12^ Unlike the steroid receptors that constitute the remainder of the NR3 subfamily that require ligands to activate transcription of target genes, the ERRs display constitutive transcriptional stimulatory activity and do not appear to require a ligand.

ERR isoforms display distinct but partially overlapping tissue distributions that align with their metabolic roles. ERRα is the most widely expressed and is particularly abundant in metabolically active tissues with high mitochondrial density, including heart, skeletal muscle, kidney, liver, intestine, and brown adipose tissue, consistent with its dominant role in oxidative metabolism and mitochondrial gene programs.^13,14^ ERRγ shows a somewhat more restricted pattern, with quite high expression in energetically demanding tissues such as heart, oxidative skeletal muscle, brain, and kidney, reflecting its strong association with mitochondrial respiration and oxidative capacity.^15–18^ In contrast to ERRα and -γ, ERRβ is comparatively limited in adult tissues and is most prominent in embryonic development, stem cells, the placenta, the ears, and the eyes, suggesting more specialized roles in development and differentiation.

### The role of ERRs in physiology and pathology and the potential for synthetic ERR agonists

ERR target genes include a network of genes within key energy-generating processes, including fatty acid oxidation (FAO), the tricarboxylic acid (TCA) cycle, mitochondrial biogenesis, and oxidative phosphorylation (OXPHOS).^19–21^ ERRs function as critical regulators of energy balance, enabling tissues to match fuel utilization with physiological demand. In the heart and skeletal muscle, ERRα and ERRγ support sustained contractile performance, exercise capacity, and resistance to fatigue by maintaining high oxidative ATP production. In metabolic tissues such as liver, adipose, and muscle, ERR activity contributes to whole-body adaptations to fasting, feeding, cold exposure, and endurance training, influencing lipid handling, glucose homeostasis, and thermogenic capacity. Impaired ERR function is associated with disrupted cardiac energetics and contributes to heart failure.^22,23^ Additionally, ERRs are implicated in metabolic disorders, where altered function influences insulin sensitivity, lipid metabolism, and the progression of conditions such as obesity, fatty liver disease, and type 2 diabetes.^10,24,25^ Thus, ERR agonists would have potential in treating these diseases.

Administration of ERR agonists in mice reproduced several exercise-like effects on systemic metabolism, including enhanced energy expenditure and increased fatty acid oxidation. These agonists induced an oxidative fiber-type switch and improved muscle endurance, suggesting potential for treating a range of muscle-function-related diseases.^26,27^ These metabolic improvements were also associated with reduced fat mass, suggesting a potential role in the treatment of obesity and in improving muscle function.^28,29^ ERR agonism holds the promise for alleviating liver metabolic diseases such as MASH^28^ and they also improved insulin sensitivity in models of metabolic syndrome.^30^ Furthermore, they alleviated heart failure (HF) symptoms by improving ejection fraction, ameliorating fibrosis, and increasing survival in pressure overload-induced HF in mouse models.^31^ Decreased mitochondrial function is a hallmark of aging,^32,33^ thus ERRs agonists may have anti-aging properties in a range of tissues. This has clearly been demonstrated in a model of reduced renal function in aged mice.^34^ There are substantial opportunities for ERRs agonists in the treatment of age-related diseases.

### Identification of ERRs agonists

As their name suggests, ERRs are homologous to the classical estrogen receptors (ERα and ERβ); however, they do not interact with endogenous estrogen receptor ligands. ERRs and ERs display considerable sequence similarity, particularly within the DBD,^9^ but unlike ERs, which rely on ligand engagement to initiate transcriptional activation, all three ERR isoforms possess inherent, ligand-independent constitutive transcriptional activity, which is dependent on relative coactivator expression level.^35^ Additionally, ERRs bind to DNA response elements that differ from the canonical palindromic estrogen response elements utilized by ERs and thus display a very distinct range of target genes, even though there is significant similarity in their DBDs.^19^ ERRα and ERRγ share a core transcriptional program centered on mitochondrial oxidative metabolism and fatty acid oxidation, with PDK4 and CPT1B representing the most consistently supported common target genes, alongside broader overlap in PPARA-, TFAM-, and OXPHOS-associated genes.^22,36,37^ However, ERRβ has distinct target genes in trophoblast stem cells, including CDX2, EOMES, SOX2, FGFR4, and BMP4.^38^

**Fig. 2:**
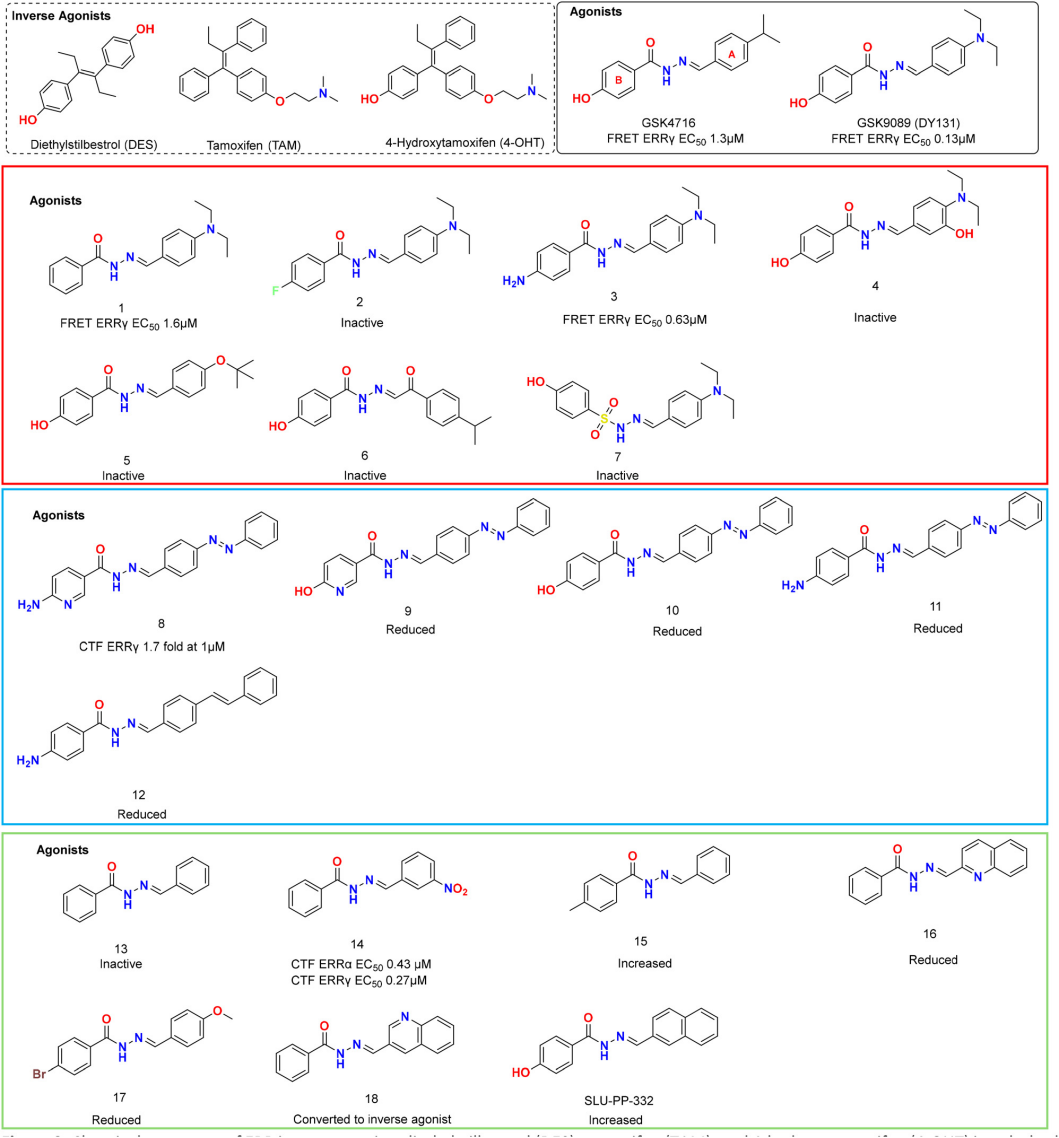
Chemical structures of ERR inverse agonists diethylstilbestrol (DES), tamoxifen (TAM), and 4-hydroxytamoxifen (4-OHT) in a dashed-line box, and acyl hydrazone-based ERR agonists. Each agonist set is framed in a separate box. The activity of the analogs was compared with that of the hit compound in each set. In each box, activity of a prototypical ligand is illustrated and analogs are shown with activity that is either reduced or increased as indicate.

Prior to 2002, no small molecule ERR ligands had been identified. Searching for ERRs ligands, Coward *et al.* tested a library of ER ligands, including synthetic ER ligands such as diethylstilbestrol (DES), tamoxifen (TAM), and 4-hydroxytamoxifen (4-OHT) (Fig. 2) for disruption of the ERRγ–SRC-1.2 interaction using a fluorescence resonance energy transfer (FRET) assay. As per ERRγ, the EC_50_ values for DES, TAM, and 4-OHT were 700 nM, 400 nM, and 50 nM, respectively. The three compounds have EC_50_ values 700 nM, 950 nM, and 150 nM, respectively, on ERRβ. In contrast, on ERRα, activity was detected only with DES and was minimal (EC50 = 10 µM). The authors reproduced the results in a radioligand binding assay (RLBA), using [^3^H]4-OHT as the radioligand, which has a *K*_d_ value of 35 nM for ERRγ. DES and TAM displaced the radioligand with *K*_i_ values of 870 nM, while 4-OHT *K*_i_ value was 75 nM. In a co-transfection assay in CV-1 cells using an ERRγ expression plasmid, these compounds functioned as inverse agonists, with 4-OHT the most active, substantially decreasing ERRγ transcriptional activity.^39^

Although a range of other ERR inverse agonists (IA) would be identified targeting each of the three ERRs, including ERRα IA XCT790,^40^ ERRα/γ IA SLU-PP1072,^13^ ERRα IA C29,^41^ and ERRγ IA GSK5182,^41^ Identification of ERR agonists was considerably more challenging. Biochemical coactivator interaction assays demonstrated that ERRs constitutively associate with transcriptional coactivators, consistent with their ligand-independent activity. These findings suggested that the AF-2 domain adopts an activation-competent conformation in the absence of ligand. Consequently, this raised the possibility that pharmacological ligands might not further enhance ERR transcriptional activity.

### Acyl hydrazone derivative ERR agonists

The first selective ERR agonists discovered were selective for ERRβ and ERRγ and were phenolic acyl hydrazone derivatives (GSK4716 and GSK9089 (Synonym: DY131)) (Fig. 2), which bound and activated these two receptors with selectivity over the estrogen receptors (ERs).^42,43^ In a FRET assay, GSK4716 and GSK9089 activated ERRγ as indicated by a 40–55% increase in coactivator peptide recruitment with respective EC_50_ of 1.3 µM and 0.13 µM. Surprisingly, in a luciferase assay in HeLa cells, GSK4716 showed significant activity at 1 µM, whereas GSK9089 was less potent in the luciferase assay, requiring 10 µM (Table 1).

**Table 1:** Acyl hydrazone class ERRs agonists

Compound	Affinity (nm)		Luciferase assay (µM)		FRET (µM)		Notes
	ERRα	ERRγ	ERRα	ERRγ	ERRα	ERRγ	
GSK4716^42^	—a	2 × 10^3^	i.a.	Active at 1 µM	i.a.^b^	1.3	(1) GSK4716 and GSK9089 have no affinity for ERα and ERβ at concentrations up to 50 µM
GSK9089^42^	—	660	i.a.	Active at 10 µM	i.a.	0.13	(2) The affinity assay evaluated competition with [3H]-4-OHT for binding to ERRγ-LBD
1^42^	—	—	—	—	—	1.6	(3) HeLa cells were used in the reporter assay
2, 4, 5, 6, and 7^42^	—	—	—	—	—	i.a. at 3 µM	(4) A discrepancy exists between the biochemical and cellular assay results for compounds GSK4716 and GSK9089
3^42^	—	—	—	—	—	0.63	
8^44^	—	—	i.a.	∼1.7 fold	—	—	(1) The transcription activity was measured in COS-1 cells at 1 µM
9^44^	—	—	—	∼1.3 fold	—	—	(2) There are no dose–response curves provided due to testing the compounds at one point
10^44^	—	—	—	∼1.2 fold	—	—	
11^44^	—	—	—	∼1.2 fold	—	—	
12^44^	—	—	—	∼1.5 fold	—	—	
13^27^	—	—	i.a.	i.a.	—	—	(1) The co-transfection assay was performed in HEK293 cells
14^27^	—	—	0.43	0.27	—	—	(2) The gene expression was conducted using C2C12 muscle cells
15^27^	—	—	0.21	0.2	—	—	(3) The compounds lack ERR-type selectivity
16^27^	—	—	0.49	0.83	—	—	(4) All compounds showed no significant cytotoxicity
17^27^	—	—	0.54	0.40	—	—	
SLU-PP-332^29,30^	—	—	0.1	0.43	—	—	(1) It is a pan ERR agonist
							(2) It has significant exercise mimetic activity *in vivo*

a Not tested.

b i.a. inactive.

The optimal substituents on the A-ring (Fig. 2) were 4-isopropyl and 4-*N*,*N*-diethyl as identified by ERRγ FRET assay. SAR studies showed that introducing a polar or bulky hydrophobic group in this position decreases activity. Phenolic substitution is crucial for activity, as testing the unsubstituted B-ring analogue of GSK9089, compound 1, showed reduced activity, whereas 4-fluoro substitution completely abolished activity, as in compound 2. Notably, the introduction of the 4-NH_2_ group in compound 3 retained activity, indicating that the –NH_2_ group worked well as a bioisostere of the –OH group, forming the same H-bonds. That is logical because the phenolic ring of estradiol forms a H-bond with Glu and Arg residues of ERs, which are conserved in ERRs. Furthermore, polar substitution or bulky nonpolar substitution on the A-ring abolished activity, as in compounds 4 and 5. Remarkably, the analogous diacyl hydrazine (6) and sulfonyl hydrazone-based derivative (7) were inactive in this assay. The ERRγ FRET results for GSK4716 and GSK9089 were consistent with those from an independent radioligand competition binding assay with respective IC_50_ of 2 µM and 660 nM. Both compounds showed no activity against the classical ERs at concentrations up to 50 µM, making them the first selective ERR agonists.^42^

Yu *et al.* further explored the agonist activity of GSK9089 on different ERR isoforms.^43^ In CV-1 cells, ERRs activity was tested with a GAL4 DBD reporter construct and fusion proteins containing the LBD of the three types of ERR linked to the DBD of the yeast GAL4 protein in the presence of GSK9089. Notably, the GAL4 DBD assay is a reporter assay used to evaluate the transcriptional activity of a protein domain, commonly the ligand-binding domain of a nuclear receptor. In this system, the domain of interest is fused to the GAL4 DBD, and the resulting fusion protein binds to GAL4 response elements placed upstream of a reporter gene such as luciferase. When a test compound modulates the activity of the fused domain, the reporter signal changes accordingly, enabling assessment of agonist, antagonist, or inverse agonist effects in a simplified, controlled transcriptional context.^45^

Activation of ERs was also examined to confirm the selectivity of GSK9089. Notably, ERRα showed no sensitivity to GSK9089 across concentrations, consistent with previous data. Conversely, ERRβ transcriptional activity was induced three- to four-fold at concentrations of 10–30 µM, while ERRγ exhibited a 6.6-fold activation at 30 µM. As anticipated, GSK9089 exhibited no activating or inhibitory effects on either ERα or ERβ in this study.^43^ Collectively, those results identified GSK9089 and GSK4716 as beneficial lead compounds for probing ERRγ pharmacology.^46–48^

### X-ray co-crystal structure of ERRγ-LBD and GSK4716

Structural studies confirmed that ERRs adopt an active conformation in the absence of ligand, providing a molecular basis for their constitutive transcriptional activity. To explore the binding mode of GSK4716 into ERRγ to be used in structure-based drug design, Wange *et al.* co-crystallized GSK4716 onto ERRγ-LBD.^9^ X-ray crystal structures of the ERRs-LBD in three functional states: unliganded, agonist-bound, and inverse agonist-bound, reveal the molecular basis of small-molecule regulation, providing the first high-resolution structural insight into the unique regulatory mechanisms of ERRs.

**Fig. 3:**
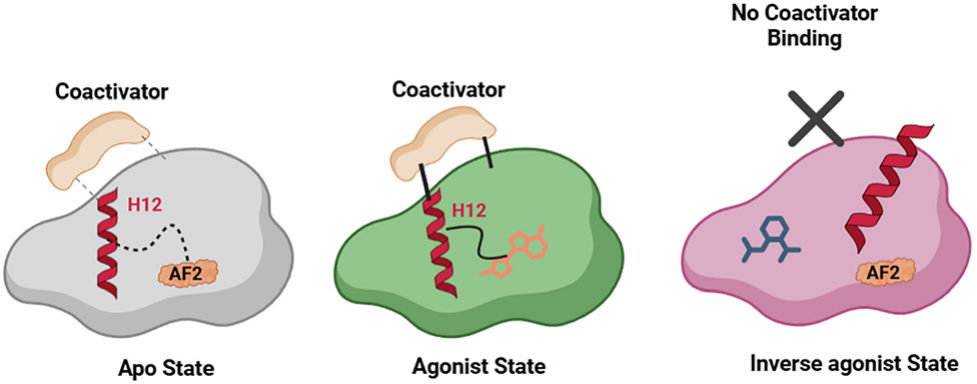
Schematic representation of ERRγ ligand-binding domain conformations in apo, agonist-bound, and inverse agonist-bound states, adapted conceptually from crystallographic studies. Helix 12 is maintained in a conformation competent for coactivator interaction in the apo receptor state but can be stabilized into a more efficient state upon agonist binding. When inverse agonists are bound the subsequent conformational changes, leading to repositioning of H12 in an inefficient state that does not allow for effective interaction with coactivators.

In contrast to classical ERs, and as expected given their constitutive transcriptional activation activity, ERRs adopt an active conformation in the absence of ligand, with H12 pre-positioned to support constitutive coactivator recruitment. Structural comparison of the apo receptor, agonist-bound, and inverse agonist-bound states demonstrated that small molecules modulate ERR activity primarily through stabilization or displacement of H12 within the activation function-2 (AF-2) surface. The GSK4716 agonist was shown to stabilize active conformation and enhance coactivator binding, as demonstrated by thermal stability assay.^9^ In contrast, the inverse agonist 4-hydroxytamoxifen induced H12 repositioning, disrupting the AF-2 interface and suppressing basal transcriptional activity, with no significant conformational changes in helices 1–10 (Fig. 3). These findings established the molecular framework underlying small-molecule regulation of ERRs and provided a structural rationale for the divergent pharmacological behaviors observed among ERR agonists and inverse agonists.^9^

**Fig. 4:**
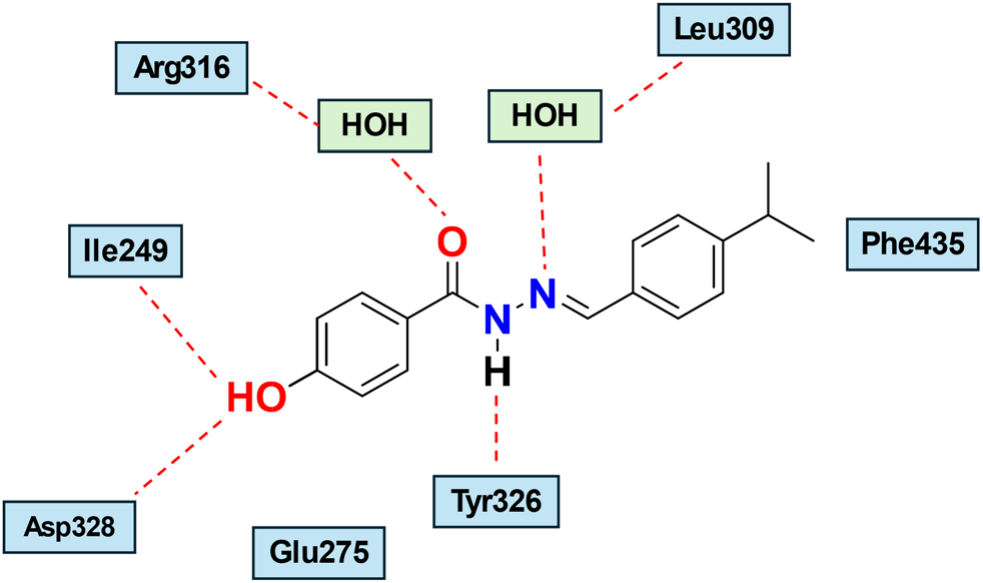
Schematic representation of the binding mode of GSK4716 interaction within ERRγ (PDB:2GPP). Hydrogen bonds involving the ligand and amino acid residues or water molecules are shown in red dashed lines.^9^

Notably, most amino acid residues that form the ERRγ ligand binding pocket (LBP) are conserved with ERα. However, subtle substitutions at several corresponding positions reduce the overall volume of the LBP. The main difference is ERR Phe-435, which corresponds to ERα Leu-525 and defines one side of the ERRγ pocket, preventing ERRγ from accommodating bulky steroidal estrogens. The volume of apo-ERRγ LBD is 280 Å,^3^ while it is 480 Å^3^ for ERα, justifying the selectivity of bulky steroidal estrogens to ERs without affecting ERRs.^9^Fig. 4 provides a schematic representation of the interaction between GSK4716 and ERRγ-LBD.

**Fig. 5:**
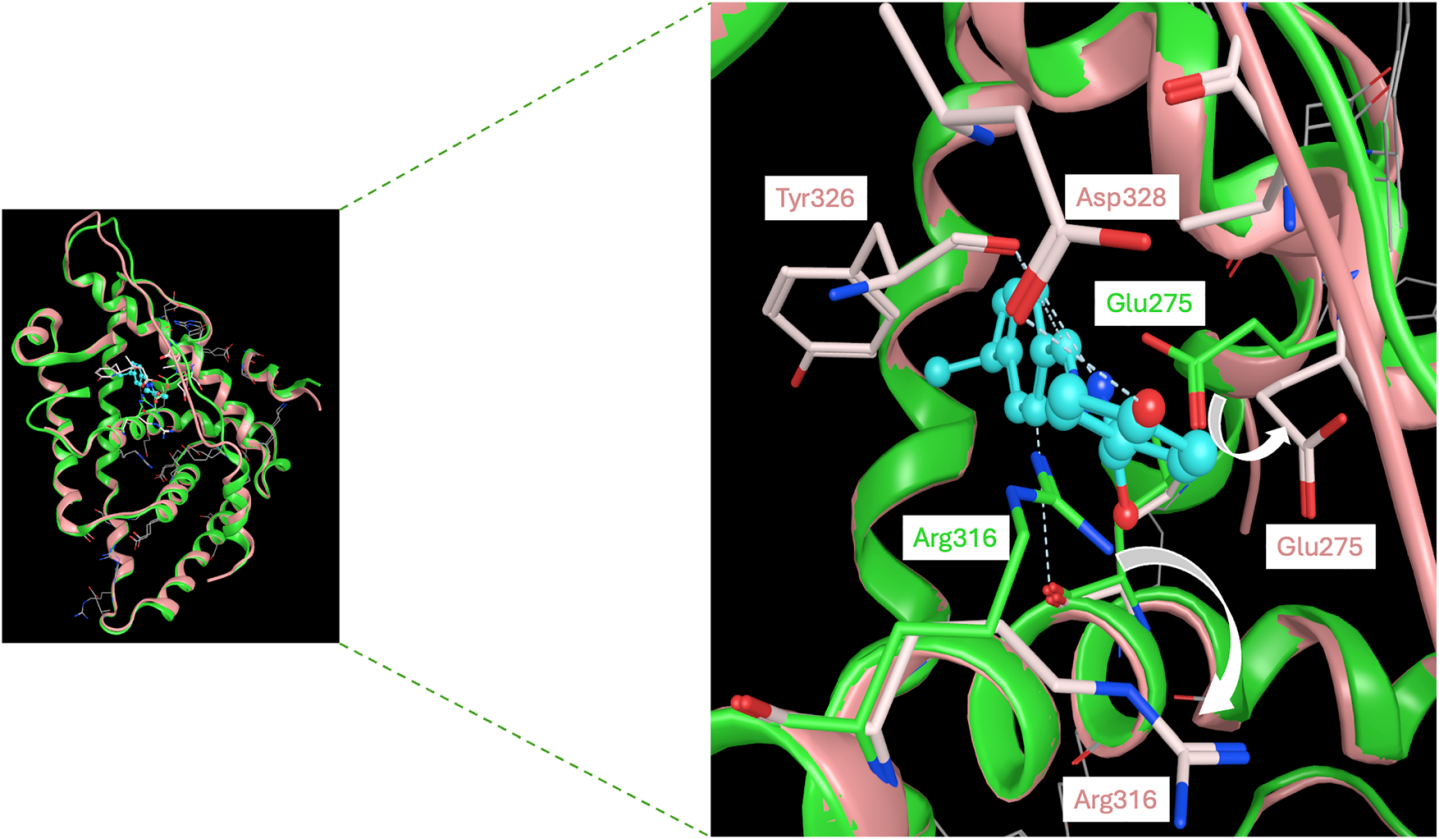
Left: A zoom-out view of superimposed apo-ERRγ (PDB:2GPO) with green ribbons and green stick amino acid residues, and holo-ERRγ (PDB:2GPP) in pink ribbons and pink stick amino acid residues co-crystalized with a synthetic agonist GSK4716 shown in cyan ball and stick. Right: A zoom-out view of the LBP demonstrating the H-bond interaction between GSK4716 and Tyr326 residues and Asp328, leading to its induced-fit, creating a larger merged pocket through shifting Glu275 and Arg316, as indicated by the white solid curved arrows.^9^

Here, we will dissect the binding mode of GSK4716 to the ERRγ-LBD (PDB: 2GPP) to rationalize its selectivity over the classic ERs (Fig. 5). The pivotal phenolic group of GSK4716 forms a hydrogen bond with Asp328 and Ile249.^9^ In ERα, Asp328 corresponds to Pro406, which could not interact in the same way with the phenolic group.^49^ The carbonyl of the acyl hydrazone bridges to two water molecules, which in turn interact with Arg316 and Leu309. Interestingly, the co-crystal structure demonstrated that GSK4716 induces a conformational rotation of both Glu275 and Arg316, thereby enabling the ligand to access an additional cavity within the ligand-binding domain (LBD). The resulting merged pocket, with a total volume of approximately 610 Å^3^, is sufficiently spacious to accommodate the acyl hydrazone ligand without necessitating displacement of the AF-2 helix, in contrast to what is observed with inverse agonists.

Kim *et al.* discovered more potent ERRγ agonists than GSK4716, exhibiting excellent selectivity over ERRα and ERRβ, through a rational design approach combined with biomedical analysis and computational docking simulations.^44^ The authors conducted an extensive SAR study of the acyl hydrazone scaffold by synthesizing a 30-compound library. The compounds were benchmarked using a cell-based reporter-gene assay to assess their effects on ERRγ transcriptional activity. They used a one-hybrid GAL4 fusion assay in COS-1 cells, transiently transfecting a vector expressing the LBD of ERRγ fused to the DBD, along with a reporter plasmid containing five GAL4 binding sites upstream of the firefly luciferase reporter gene. At 1 µM concentration, many hits outperformed GSK4716 in enhancing ERRγ transcriptional activity in the reporter assay (Table 1).

Of note, the design of some compounds was based on introducing a bulky phenyldiazenyl benzylidene motif to enable them to occupy both merged pockets through induced fit. This conferred compound 8 high ERRγ activation and a selectivity over ERRα/β. Compound 8 promoted ERRγ transcriptional activity, even at a 10 nM concentration, and its efficacy at this concentration was comparable to GSK4716 at 1 µM concentration. A docking study of this compound within the ERRγ LBD suggested that it stretches between the two merged pockets, as anticipated. From the SAR standpoint, the 4-amino substitution showed better performance than the 4-OH (9), and the pyridine ring is more effective than the benzene ring (10) and (11). Finally, styryl benzylidene (12) demonstrated lower activity than phenyldiazenyl benzylidene. The limitation of this work is the lack of an independent biochemical assay. Furthermore, the activity was tested at a single concentration, which prevented the generation of dose–response curves and resulted in less reliable data for fully understanding the compounds' potency.^44^

Given the prominent role of ERRα in mitochondrial function and its potential as a drug target, we focused on identifying ERRα agonists. The initial successes in identifying ERR and ERR agonists suggested an opportunity, but ERR's LBP is smaller and more restrictive than ERR or −, leading to suggestions that it was intractable.^50^ To this end, we conducted an extensive SAR study of GSK4716. This was achieved by retaining the hydrazone linker and modifying the aryl groups on both the left and right sides. Removal of all substitutions on the phenyl rings of GSK4716 resulted in a loss of activity, as shown by compound 13. Diversification of the substituents led to the discovery of potent ERR pan-agonists, such as compounds 14, 15, 16, and 17, which displayed sub-micromolar activity in luciferase reporter assay (Table 1). Surprisingly, minor structural modifications of some agonists converted them into inverse agonists such as compound 18 (Fig. 2).^27^

The most active compounds in this series were tested for their ability to regulate ERR target genes expression, including PGC-1α, PGC-1β, CPT1α, and PDK4. Notably, these genes are involved in cellular and tissue energy metabolism in high-energy-demand tissues. PGC-1α and PGC-1β are key drivers of mitochondrial biogenesis. CPT1α catalyzes the initial step in the transport of long-chain fatty acids into mitochondria and is considered a rate-limiting enzyme for fatty acid β-oxidation. PDK4 plays a central role in maintaining energy homeostasis by regulating metabolic flux and catalyzing the conversion of pyruvate to acetyl-CoA.^27^ At 1 µM, 14 significantly upregulated the expression of PGC-1β, CPT1α, and PDK4. Likewise, 15 significantly upregulated for all tested target genes, except CPT1α. Compound 17 increased mRNA expression of PGC-1α, PGC-1β. Interestingly, those compounds are non-cytotoxic, as shown by cell viability tests at 1 µM and 10 µM, suggesting a favorable safety profile.

Ultimately, a structure-based design strategy for GSK4716, guided by the ERRγ LBD-GSK4716 X-ray crystal structure (PDB: 2GPP), led to the identification of SLU-PP-332, an outstanding ERRs pan-agonist. SLU-PP-332 retains the phenolic ring, as in GSK4716, along with an unsubstituted naphthyl B-ring. Molecular modeling of GSK4716 within the ERRα ligand-binding domain suggested that enhancing hydrophobic and aromatic interactions, particularly with Phe328 unique to ERRα, could improve receptor affinity and efficacy. Replacement of the isopropyl phenyl substituent with a naphthalene moiety was predicted to introduce favorable π–π stacking interactions, stabilizing ligand engagement within the agonist pocket. This rational modification yielded SLU-PP-332, which displayed markedly improved ERRα potency while maintaining activity at ERRβ and ERRγ. Its EC_50_ against ERRα, ERRβ, and ERRγ in a luciferase reporter assay using HEK293 cells was 98 nM, 230 nM, and 430 nM, respectively.^29^

SLU-PP-332 displayed broad *in vivo* biological activities.^29,30^ It significantly upregulated PDK4 mRNA expression in C2C12 myoblasts at 1 and 5 µM concentrations and enhanced mitochondrial respiration. Without cytotoxic effect, administration of SLU-PP-332 in mice increased oxidative fibers in muscle cells, increased fatty acid oxidation, and enhanced exercise endurance. SLU-PP-332 has the potential to be translated into a clinical exercise mimetic that can alleviate metabolic syndrome by reducing fat mass and improving glucose metabolism in mouse models of obesity.^30^ Moreover, SLU-PP-915 markedly enhanced cardiac function by increasing ejection fraction, attenuated fibrotic remodeling, and improved overall survival in a mouse model of pressure overload-induced heart failure.^31^ Of note, SLU-PP-332 lacks oral bioavailability and is metabolically unstable as discussed below.^51^

**Fig. 6:**
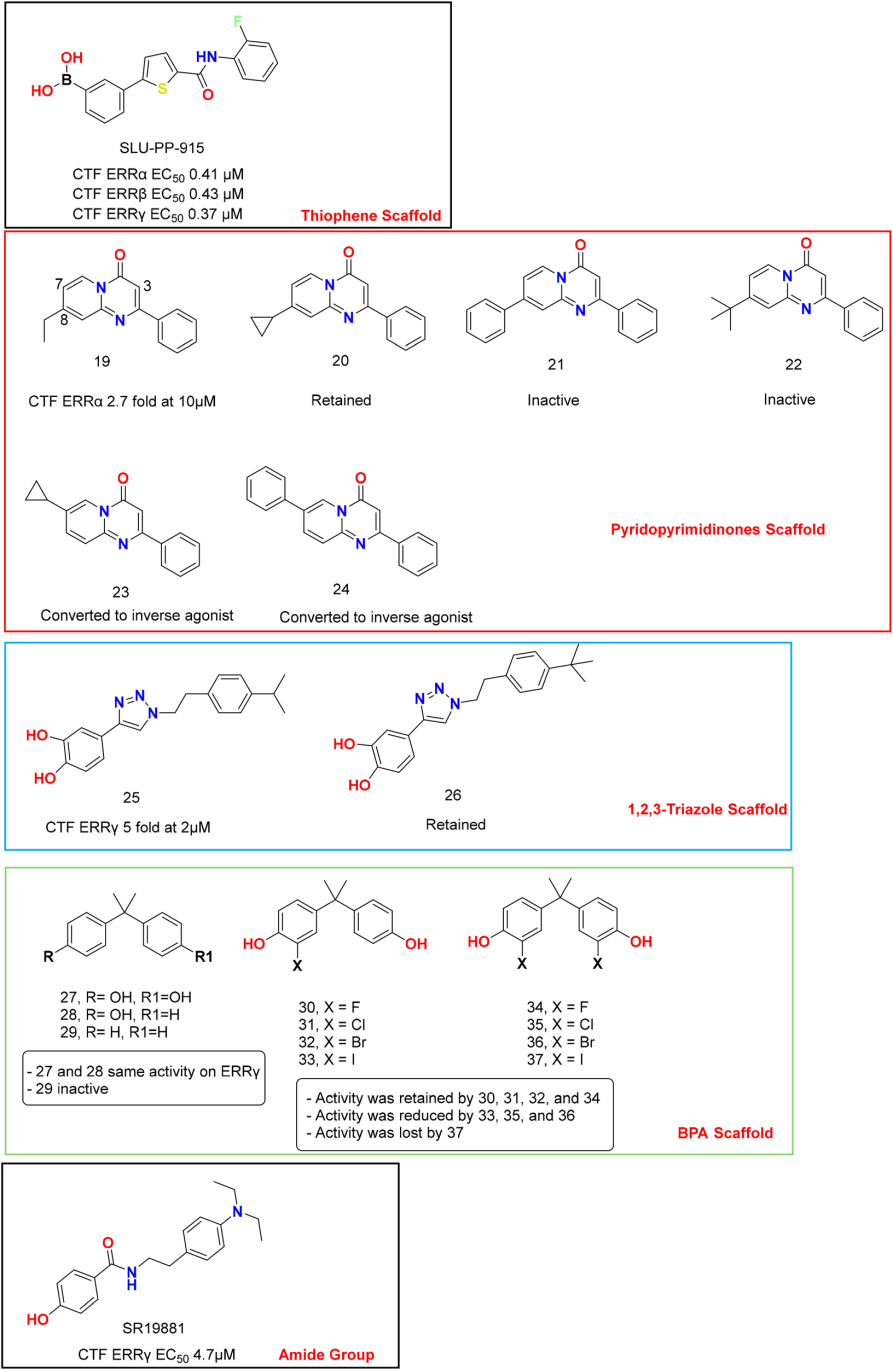
Chemical structure of ERRs ligands with diverse chemical scaffolds. Each set of compounds is framed in a separate box. The activity of the analogs was compared with that of the hit compound in each set. In each box, activity of a prototypical ligand is illustrated and analogs are shown with activity that is either reduced or increased as indicated.

Most of the ERR agonists contained hydrazide/hydrazone derivatives; however, other agonists with different scaffolds exist as discussed below (Fig. 6). It is worth noting that the hydrazide/hydrazone linkage is considered problematic in medicinal chemistry due to its rapid metabolism and possible toxicity.^52,53^ For example, GSK4716 exhibited a very short human liver microsomal half-life of 6.4 minutes, which severely limits its *in vivo* utility, prompting the replacement of the hydrazone linker.^42,54^ Though highly potent and efficacious, SLU-PP-332 is not considered metabolically stable enough to be a classic drug candidate without further optimization.

### 2,5-Disubstituted thiophenes as ERRα/β/γ

**Fig. 7:**
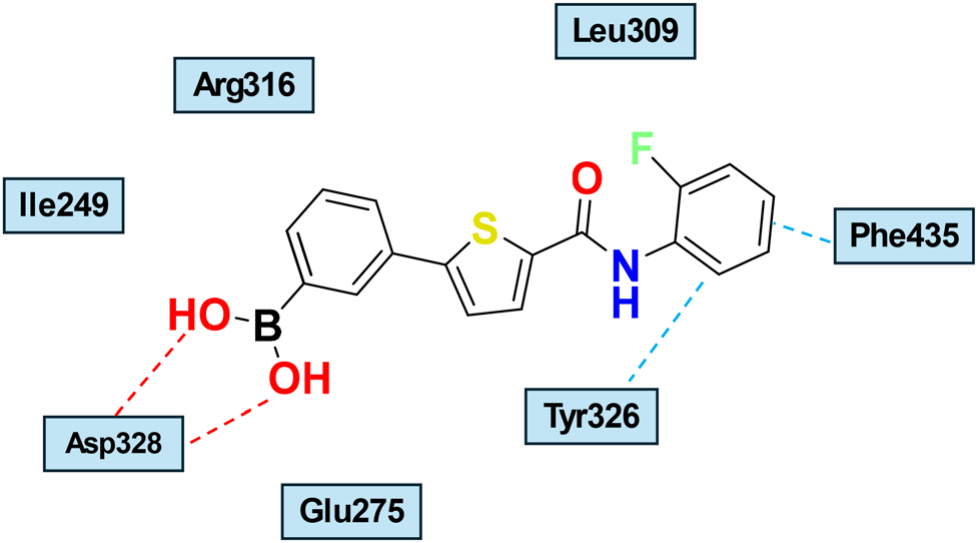
Schematic representation of the predicted binding mode of SLU-PP-915 into the crystal structure ERRγ (PDB:2GPP). Hydrogen bonds involving the ligand and amino acid residues are shown in red dashed lines. The π-stacking with Tyr326 and Phe435 is shown in blue dashed lines.^55^

We continued expanding our search for chemically diverse pan-ERR agonists by employing a thiophene carboxamide bioisostere strategy to cyclize hydrazones. This led to the identification of an effective pan-ERR agonist, SLU-PP-915, that demonstrated consistent activity at cell-based and *in vivo* activity.^55^ SLU-PP-915 has not yet been co-crystallized with ERR, but its molecular docking into ERRγ suggests that it adopts a binding mode similar to that of GSK4716, forming non-covalent interactions, including two hydrogen bonds with Asp328, π-stacking with Tyr326, and Phe435, thereby expanding the small LBP (Fig. 7). In transiently transfected cell-based luciferase reporter assays, SLU-PP-915 produced a clear concentration-dependent increase in transcriptional activity for each ERR isoform, with nanomolar EC_50_ values and near-maximal efficacy, consistent with full agonism (Table 2). Notably, the magnitude and potency of activation were well-balanced across the three receptors, indicating the absence of a pronounced isoform bias. The functional relevance of ERR activation was further confirmed in ERR-expressing cell lines, where SLU-PP-915 significantly upregulated the endogenous target genes PGC1α, LDHA, and PDK4, associated with mitochondrial biogenesis, oxidative phosphorylation, and energy metabolism. Selectivity profiling against nuclear receptors ERα and ERβ revealed minimal off-target agonist activity, underscoring the specificity of SLU-PP-915 for ERR signalling. Collectively, these data establish SLU-PP-915 as a potent, selective, and balanced pan-ERR agonist with a robust transcriptional and functional activity *in vitro*. One drawback of the SLU-PP-915 structure is the presence of an electrophilic boronic acid substitution on the A-ring, which acts as a Michael acceptor and can form covalent bonds with off-target proteins. However, using a boronic acid moiety instead of a phenolic or aniline group modestly improved metabolic stability in human and mouse microsomal *in vitro* assays. SLU-PP-915 was found to be stable in both microsomes with *T*_1/2_ > 60 min.^55^ Remarkably, SLU-PP-915 is orally bioavailable and possesses an exercise-mimetic effect *in vivo*.^51^ Both SLU-PP-915 and SLU-PP-332 significantly improved aerobic exercise capacity – measured by increased running distance and duration – when delivered intraperitoneally. Importantly, after adjusting for systemic exposure, comparable efficacy was preserved with oral administration.^51^ This was proven by a treadmill run-to-exhaustion test and by examining quadricep *Ddit4* induction in treated and untreated mice. Importantly, SLU-PP-915 displayed exercise-mimetic activity *in vivo*^51^ and efficacy in heart failure models.^31^

**Table 2:** Chemical structure of ERRs agonists with diverse chemical scaffolds

Compound	Binding (nm)		Luciferase assay (µM)		FRET		Notes
	ERRα	ERRγ	ERRα	ERRγ	ERRα (µM)	ERRγ (µM)	
SLU-PP-915^55^	—a	—	0.41	0.378	—	—	(1) The percentage of efficacy is 90% and 167% for ERRα and ERRγ, respectively, compared to SLU-PP-332
							(2) It is orally bioavailable, unlike SLU-PP-332
19^56^	—	—	2.7-fold	—	—	—	(1) The luciferase assay was performed at 10 µM
20^56^	—	—	2.6-fold	—	—	—	(2) 16 is active in a dose-dependent manner
21^56^	—	—	i.a.^b^	—	—	—	(3) Moving the same substitution from position 8 to 7 converted the ligand into an inverse agonist
22^56^	—	—	i.a.	—	—	—	
25^18^	—	46 × 10^3^	—	5-fold	—	—	(1) The binding was tested by ITC assay
26^18^	—	—	—	4-fold	—	—	(2) The reporter gene assay was made at 2 µM
							(3) In the same conditions, GSK4716 activated transcription by 1.85-fold
27^57,58^	—	13.1	—	Retain activity	—	—	(1) 24 and 25 have similar binding affinity to ERRγ, and both have similar binding mode in their co-crystal structure with ERRγ-LBD
28^58^	—	13.9	—	Retain activity	—	—	(2) In HeLa cells, 24 preserved the constitutional activity of ERRγ
29^58^	—	i.a.	—	—	—	—	
30^59^	—	5.30	—	Retain activity	—	—	(1) Monosubstituted phenyl rings of BPA work better than disubstituted and tri-substituted analogues
31^59^	—	15.1	—	—	—	—	(2) Mono-fluoro (27) and Di-fluoro (31) substituted BPA derivatives possess the highest affinity
32^59^	—	31.5	—	—	—	—	(3) 27 and 31 preserve transcriptional activity in HeLa cells
33^59^	—	282	—	—	—	—	
34^59^	—	5.95	—	Retain activity	—	—	
35^59^	—	56.6	—	—	—	—	
36^59^	—	1550	—	—	—	—	
37^59^	—	i.a.	—	—	—	—	
SR19881^54^	—	—	—	4.7	—	390	(1) It is metabolically unstable with a short *t*_1/2_ in human liver microsomal enzymes

a Not tested.

b i.a. inactive.

### Pyrido[1,2-α]pyrimidine-4-ones as ERRα agonists

The pyrido[1,2-α]pyrimidin-4-one scaffold was identified through ligand-based optimization by Peng *et al.* to agonize ERRα. The series was screened in 293FT cells by transient transfection using a vector expressing the LBD domain of human ERRα fused to the GAL4 DBD and a reporter plasmid containing the firefly luciferase gene. Compounds 19 and 20 were the most potent candidates of this series in the preliminary screening at 10 µM, denoting that using a slightly large hydrophobic substitution at position 8 promotes activity. However, using a phenyl or a *t*-butyl substitution lowered the activity, as shown by 21 and 22, respectively (Table 2). Any substitution at position 3 is detrimental to the agonistic activity of ERRα. Interestingly, when the hydrophobic group was relocated from the original 8-position to the 7-position, the resulting compounds, 23 and 24, became inverse agonists of ERRα, inhibiting its transcriptional function.

Concurrently, compound 19 enhanced ERRα transcriptional activity in a dose-dependent fashion, as demonstrated by a luciferase reporter assay. In parallel, it significantly increased both mRNA and protein expression levels of MCAD, PDK4, and ATP5b, as determined by quantitative real-time PCR and western blot analyses, respectively. Furthermore, compound 19 produced a moderate, dose-dependent enhancement of glucose and fatty acid uptake in C2C12 myoblasts.^56^

### 1,2,3-Triazoles as ERRγ agonists

In an attempt to replace the problematic hydrazide/hydrazone with a non-classical bioisostere, Xu *et al.* designed 1,2,3-triazole-based ERRγ agonists. As ERRγ agonists, compounds 25 and 26 highly upregulated ERRγ transcriptional activity by 5-fold and 4-fold, respectively, in a two-hybrid reporter gene assay, compared to 1.85-fold by GSK4726. Treatment with 25 led to a dose-dependent increase in ERRγ transcriptional activity. Concomitantly, 25 increased the expression of ERRγ targeted genes, such as SHP (Small Heterodimer Partner), ATP5b, and MCAD, at the mRNA level in a qRT-PCR assay, and increased ATP5b and MCAD protein levels in a western blot assay in a dose-dependent manner.^18^

The study included a direct binding assay between 25 and ERRγ-LBD using isothermal titration calorimetry (ITC). Surprisingly, 25 binds to the protein with a *K*_d_ value of 46 µM, about 10-fold less than GSK4726 (*K*_d_ = 5.0 µM) under the same assay conditions (Table 2). Compound 25 induced a dose-dependent upregulation of UCP1 expression in white adipocytes differentiated from mouse embryonic fibroblasts (MEFs) at both the mRNA and protein levels. This response promotes adipose tissue browning, a process characterized by enhanced mitochondrial biogenesis and remodeling, which are hallmark features of brown fat development.^60^ Indeed, 25 improved mitochondrial biogenesis and biological functions in MEF-differentiated white adipocytes, as confirmed by PCR and electron microscopy.^18^

### Bisphenols as ERRγ ligands

**Fig. 8:**
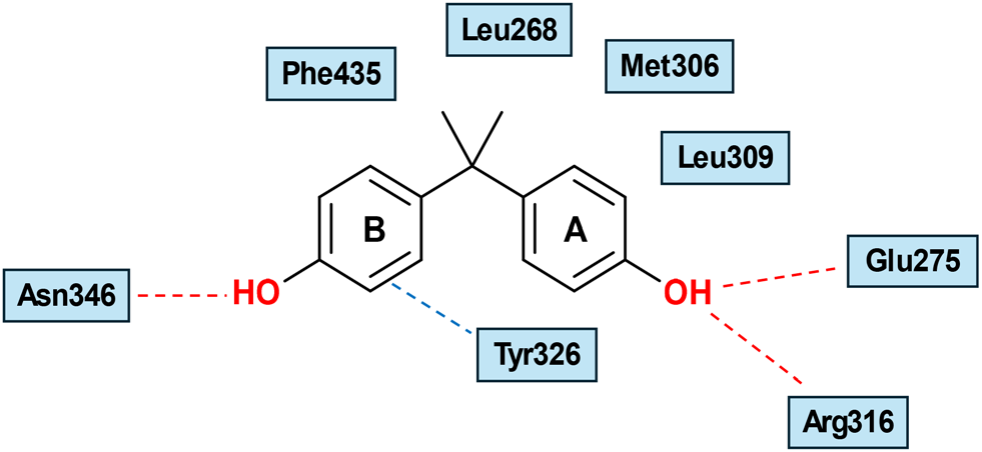
Schematic representation of BPA (27) binding mode into ERRγ-LBD (PDB: 2E2R).^61^ Hydrogen bonds involving the ligand and amino acid residues are shown in red dashed lines. The OH/π interaction with Tyr326 is shown in blue dashed lines.

Bisphenol A (BPA) (27) was found to be a strong binder to ERRγ in a RLBA, with an IC_50_ of 13.1 nM as reported by Takayanagi *et al.*^57^ Notably, Bisphenol A is a partially selective ERRγ ligand, as it has weaker affinities for ERs, ERα (IC_50_ = 1040 nM), and ERβ (IC_50_ = 1320 nM), and it is deemed an endocrine disrupting chemical (EDC). Owing to its modest binding activity to ERs, the specific mechanism of endocrine disruption by BPA remains controversial. Examination of reporter gene activity of BPA in HeLa cells co-transfected with an ERRγ expression plasmid revealed retained basal constitutive activity. BPA reversed the inverse activity of 4-OHT in a dose-dependent manner.^57^ The X-ray structure of ERRγ co-crystalized with BPA (PDB: 2E2R) showed that it binds to the receptor cavity without changing any internal structures of the pocket of the ERRγ-LBD apo form.^61^ The two phenolic -OHs are arranged to cross-link between Glu275/Arg316 and Asn346. A-ring is sandwiched between Leu309 and Tyr326 by characteristic hydrophobic interactions, while B-ring is in proximity to Tyr326, forming an OH/π interaction, which helps anchor BPA in the LBD of ERRγ (Fig. 8). BPA can preserve receptor constitutive activity by maintaining helix 12 in an active conformation.^61^ Of note, simultaneous mutation to Ala at positions 275 and 316 resulted in an absolute inability to adopt BPA in ERRγ LBD. Understandably, with the polypharmacology of BPA (it also targets a range of additional NRs and other biological receptors as well), severe toxicity is associated with this compound, including cancer, hormonal disruption, immunosuppression, infertility, and obesity.^62^

**Fig. 9:**
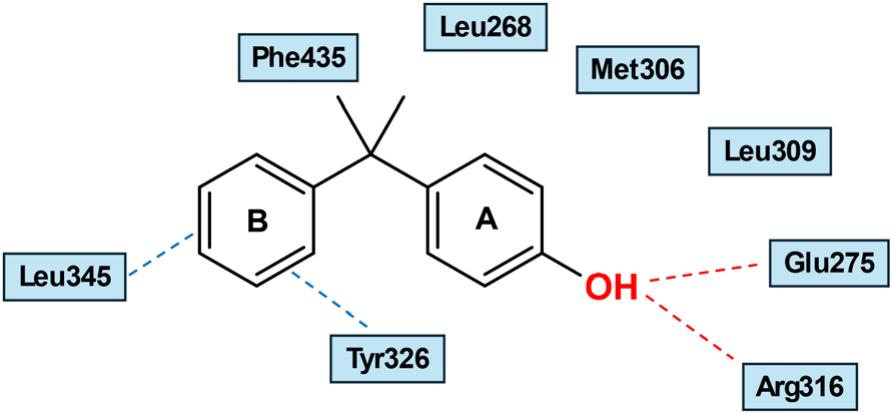
Schematic representation of compound 28 binding mode into ERRγ-LBD (PDB: 2ZAS). Hydrogen bonds involving the ligand and amino acid residues are shown in red dashed lines. The OH/π and CH-π interactions with Tyr326 and Leu345, respectively, are shown in blue dashed lines.

**Fig. 10:**
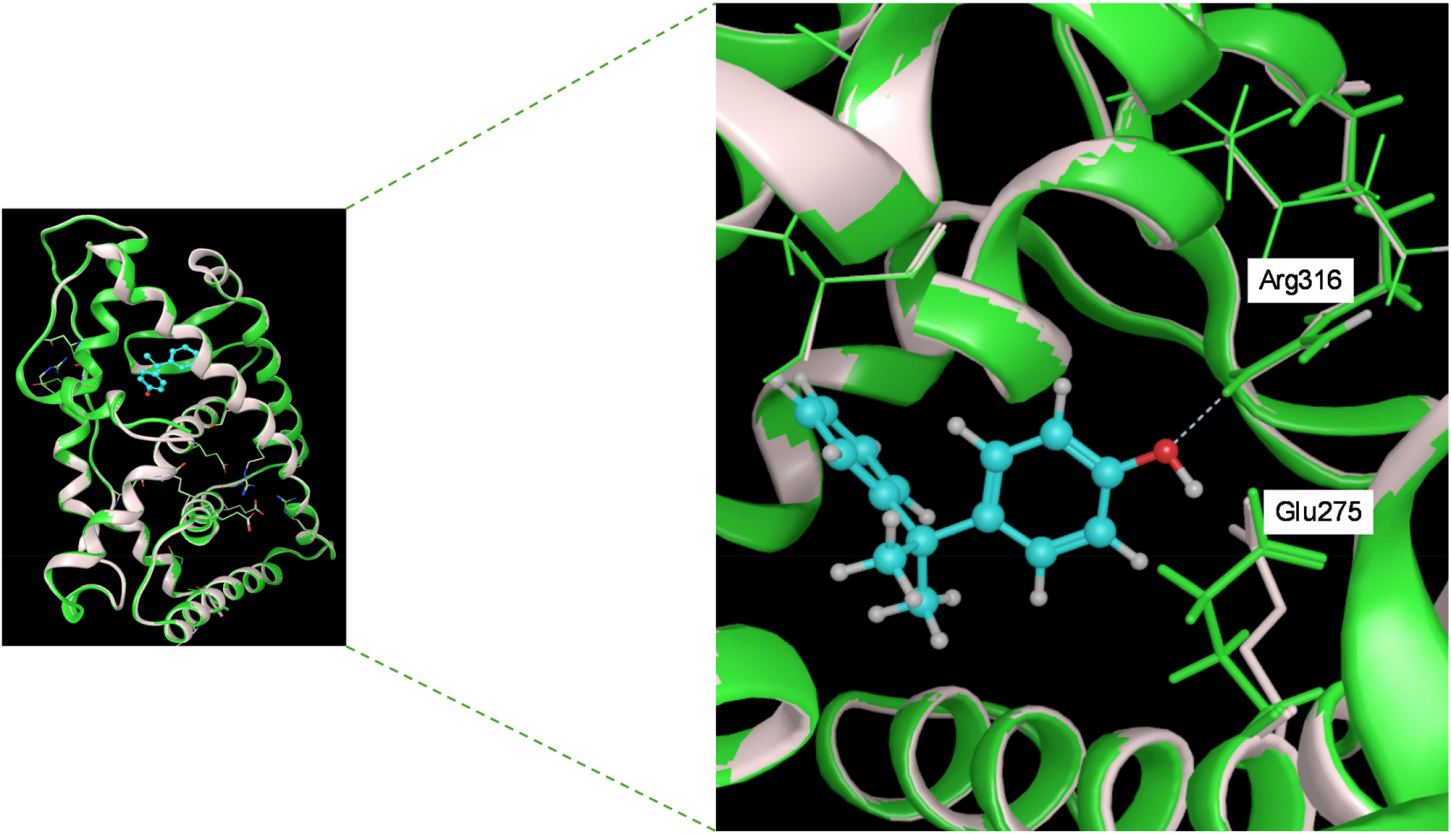
Left: A zoom-out view of superimposed apo-ERRγ (PDB:2ZBS) with green ribbons and green stick amino acid residues, and holo-ERRγ (PDB:2ZAS) in off-white ribbons and off-white stick amino acid residues co-crystalized with a synthetic agonist compound 28 shown in cyan ball and stick. Right: A zoom-out view of the 3D placement of compound 28 into the LBP binding to Glu275 and Arg316 gatekeepers without changing their conformation or creating a merged pocket.^58^

Matsushima *et al.* investigated the SAR of BPA by removing one phenolic or both phenolic –OHs, as shown by 4-α-cumylphenol (28) and 2,2-diphenylpropane (29), respectively (Fig. 6). Intriguingly, 28 is as potent as 27, while 29 was inactive in RLBA, denoting that the presence of one phenolic –OH is crucial for binding. X-ray crystallography (PDB: 2ZAS) revealed that 28 adopts a similar binding mode to BPA, lacking one H bond with Asn346, which is compensated by a CH-π interaction with Leu345 (Fig. 9).^58^ Like BPA (27), compound 28 did not induce conformational changes in the gatekeepers Glu275 and Arg316, occupying the small LBP without creating a larger merged pocket, unlike GSK4716. The titled amino acid residues in both the apo- and holo-ERRγ were almost superimposed (Fig. 10).

Another SAR study aimed at the synthesis and evaluation of next-generation BPAs was conducted by Suyama *et al.*, who introduced diverse halogen substitutions onto the two benzene rings of BPA (Fig. 6). RLBA of compounds 30, 31, 32, and 33 with monosubstituted BPA on ERRγ showed IC_50_ values of 5.3 nM, 15.1 nM, 31.5 nM, and 282 nM, respectively. This indicates that halogen substitution of a smaller size results in a higher affinity for ERRγ. However, the IC_50_ values of disubstituted BPA derivatives 34, 35, and 36 were calculated to be 5.95 nM, 56.6 nM, and 1.55 µM with a similar activity pattern to the monosubstituted congeners (Table 2). This indicates that 30 and 34 are stronger than the parent BPA. Furthermore, compound 37, with a diiodo substitution, showed no affinity for ERRγ-LBD, indicating that bulkier substituents cause significant atomic clashes and interrupt binding.

Convsersely, tri- and tetra-substituted BPA halogenated derivatives displayed lower affinity, as anticipated. Active compounds in RLBA were tested to evaluate their effects on ERRγ transcriptional activity in HeLa cells using a reporter gene assay. As with BPA, they did not activate ERRγ; rather, they only maintained its constitutive activity. Compounds 27 and 31 inhibited the binding of 4-OHT inverse agonist, thereby maintaining ERRγ's high transcriptional activity. These types of ligands, which suppress inverse agonists, are sometimes called “inverse antagonists”.^59^ Compounds 27 and 31 showed inverse antagonism, with EC50 values of 1.58 nM and 4.3 µM, respectively. The X-ray crystal structure of the 27/ERRγ-LBD complex (PDB: 6K3N) was determined, revealing that compound 27 and BPA occupy the same binding site and exhibit a comparable interaction profile within the ERRγ ligand-binding pocket. One hydroxyl group of 27 established hydrogen bonds with Glu275 and Arg316, while the second hydroxyl group formed a hydrogen bond with Asn346, mirroring the interactions observed for BPA (PDB: 2E2R). Additionally, the distinctive fluorine atom of 27 was oriented toward a cavity defined by Leu268, Tyr326, Leu342, and Asn346, with primary interaction occurring at Asn346.

### Amides as ERRβ/γ agonists

Lin *et al.* focused primarily on developing simple phenol amides as ERRγ agonists, rather than using acyl hydrazones.^54^ Their strategy was to keep the phenolic headgroup, which is crucial for receptor engagement, and replace the more problematic linkage with simpler amide chemotypes. Their lead compound SR19881, the amide analogue of GSK9089, has an ERRγ EC_50_ of 0.39 µM in a binding assay and 4.7 µM in a cell-based assay (Table 2). It has equipotency on ERRβ, making it a potent dual ERRβ/γ agonist. However, it contains a phenethyl moiety, which is a metabolically soft site, and its *T*_1/2_ in human liver microsomes is 3.1 minutes, about half that of GSK4716's (∼6.4 minutes). Thus, this compound did not successfully address the metabolic liability of acyl hydrazone derivatives.^54^

## Conclusions

This review examines the chemical and pharmacological landscape of ERRs agonists, with particular emphasis on medicinal chemistry efforts directed toward their optimization. Compounds are organized by structural class, and the structure–activity relationships associated with each scaffold are critically evaluated, outlining their mechanistic advantages and pharmacological constraints. Representative chemotypes include hydrazones, substituted thiophenes, pyridopyrimidinone derivatives, triazole-containing molecules, bisphenol analogs, and amide-based frameworks. Our SAR conclusion identifies the phenolic hydroxyl group as a common-feature pharmacophore participating in a crucial binding with Asp328, as in GSK4716 and SLU-PP-915, or Glu275 and Arg316, as in BPA (27) and 28. Remarkably, compound 29, an analog of 27 and 28 without a phenolic –OH, is inactive. It is noteworthy to mention that the boronic acid group in SLU-PP-915 plays the role of the phenolic –OH. It is anticipated that this group is not involved in covalent bond formation within the ERRγ-LBP complex. The phenolic –OH group is also present in the highly active compounds GSK9089 and SLU-PP-332. The –NH_2_ group can play the same role as the –OH group, as exemplified by the two active compounds 10 and 11. Furthermore, binding to Tyr326 is observed with various agonists, including GSK4716, SLU-PP-915, 27, and 28. We highlighted that small agonists, such as compounds 27 and 28, don’t induce conformational changes in ERRγ's LBP, unlike slightly bulkier agonists, such as GSK4716 and SLU-PP-915, which form a larger merged pocket by efficiently shifting Glu275 and Arg316.

We believe that SLU-PP-915 is one of the most interesting preclinical candidates. Further structure–activity relationship studies are urgently needed. For example, using non-classical bioisosteres of the amide group, such as urea, oxadiazoles, and triazoles, should be considered.^63^ Replacing the Michael acceptor boronic acid group with a tetrazole or a carboxylic acid can reproduce the activity without the potential formation of non-specific reversible covalent bonds. Cyclic hemiboronic acids, such as benzoxaboroles, are worth considering owing to their lower toxicity and improved drug-likeness.^64,65^

Together, these analyses establish a strategic foundation for the development of new ERRs activators with enhanced efficacy and translational promise. Such compounds have the potential for treatment of a range of diseases such as heart failure, metabolic diseases (obesity, type 2 diabetes, MASH, *etc.*), dementia, and sarcopenia.

## Author contributions

Conceptualization, T. P. B.; writing original draft, M. O. R.; review and editing, M. O. R., S. G., and T. P. B.; supervision, T. P. B.; project administration, T. P. B. All authors discussed, edited, and approved the final version.

## Conflicts of interest

There are no conflicts to declare.

## Data Availability

There is no novel data associated with this review article.
